# circRNA-SFMBT2 orchestrates ERα activation to drive tamoxifen resistance in breast cancer cells

**DOI:** 10.1038/s41419-023-06006-5

**Published:** 2023-07-31

**Authors:** Zheng Li, Yaming Li, Dianwen Han, Xiaolong Wang, Chen Li, Tong Chen, Wenhao Li, Yiran Liang, Dan Luo, Bing Chen, Lijuan Wang, Wenjing Zhao, Qifeng Yang

**Affiliations:** 1 Department of Breast Surgery, General Surgery, Qilu Hospital of Shandong University, Jinan, Shandong China; 2 Shandong Cancer Hospital and Institute, Shandong First Medical University and Shandong Academy of Medical Sciences, Jinan, Shandong China; 3 Pathology Tissue Bank, Qilu Hospital of Shandong University, Jinan, Shandong China; 4 Research Institute of Breast Cancer, Shandong University, Jinan, Shandong China

**Keywords:** Breast cancer, Breast cancer, Non-coding RNAs

## Abstract

Dysregulated ERα signaling is responsible for endocrine resistance and eventual relapse in patients with estrogen receptor-positive (ER^+^) breast cancer. Thus, identifying novel ERα regulators is necessary to fully understand the mechanisms of endocrine resistance. Here, we identified circRNA-SFMBT2 to be highly expressed in ER^+^ breast cancer cells in comparison to ER^−^ cells and found that high circRNA-SFMBT2 levels were related to larger tumor size and poor prognosis in patients with ER^+^ breast cancer. In vitro and in vivo experiments confirmed that the circRNA-SFMBT2 level was positively correlated with the ERα protein level, implying a regulatory role for circRNA-SFMBT2 in ERα signaling. Moreover, we found that circRNA-SFMBT2 biogenesis could be facilitated via RNA-binding protein quaking (QKI), and biologically elevated circRNA-SFMBT2 expression promoted cell growth and tamoxifen resistance in ER^+^ breast cancer. Mechanistically, circRNA-SFMBT2 exhibits a specific tertiary structure that endows it with a high binding affinity for ERα and allows it to interact with the AF2 and DBD domains of ERα, enforcing recruitment of RNF181 to the AF1 domain of ERα. Furthermore, the circRNA-SFMBT2/RNF181 axis differentially regulated K48-linked and K63-linked ubiquitination of ERα to enhance ERα stability, resulting in increased expression of ERα target genes and tumor progression. In summary, circRNA-SFMBT2 is an important regulator of ERα signaling, and antagonizing circRNA-SFMBT2 expression may constitute a potential therapeutic strategy for breast cancer.

## Introduction

Estrogen receptor-positive (ER^+^) breast cancer accounts for two-thirds of all breast cancer cases worldwide, making it one of the most common malignancies [1]. The selective ER modulator tamoxifen can antagonize ERα transcriptional activation to control breast cancer progression by competitively inhibiting estrogen binding and is regarded as a primary option for endocrine therapy in ER^+^ breast cancer [2]. Although tamoxifen has resulted in encouraging outcomes for breast cancer patients, many treated patients will develop drug resistance and ultimately experience relapse; thus, tamoxifen resistance represents a major problem in breast cancer therapy [3, 4].

Clinical investigations have shown that functional ERα still exists in most breast cancer patients with endocrine resistance, while ESR1 mutations were found to be associated primarily with aromatase inhibitor resistance rather than tamoxifen resistance, implying a potential role for ERα in the development of tamoxifen resistance [5]. ERα belongs to a class of ligand-dependent transcription factors activated by estrogen binding and is characterized by a typical nuclear receptor structure consisting of an AF1 domain, a DNA-binding domain (DBD), and a ligand-binding domain (LBD) [6, 7]. In general, the AF1 domain mediates ligand-independent cell growth, whereas the AF2 domain located in the LBD controls ligand-dependent cell growth. In addition, the DBD is a ligand-independent domain and mediates the interaction between ERα and an estrogen response element (ERE) [8, 9]. Notably, activation of ligand-independent ERα can circumvent tumor reliance on the ligand estrogen, facilitating the development of endocrine resistance [10]. In addition, a substantial number of studies have suggested that posttranslational modifications of ERα, such as ERα ubiquitination and acetylation, are strongly correlated with breast cancer progression and endocrine resistance [11]. For example, RNF181 promotes breast cancer progression by enhancing K63-linked ubiquitination and stabilization of ERα [12]. TRIM56 exerts similar effects by interacting with the AF1 domain of ERα in breast cancer [13]. The LIM protein Ajuba can target DBC1 and CBP/p300 for acetylation of ERα to enhance the expression of ERα target genes and induce tamoxifen resistance in breast cancer cells [8]. Given that dysregulation of ERα signaling is responsible for breast cancer progression and endocrine resistance, further investigation of new regulators of ERα signaling will facilitate the development of more effective therapeutic strategies to overcome endocrine resistance and inhibit tumor progression.

Circular RNAs (circRNAs) are a novel class of noncoding RNAs generated from precursor mRNAs (pre-mRNAs) by backsplicing of exons in eukaryotic genomes [14–16]. The process of circRNA biogenesis can be facilitated or inhibited by some RNA-binding proteins, such as Quaking (QKI) [17], Fused in Sarcoma (FUS) [18], and DExH-box helicase 9 (DHX9) [19]. Importantly, numerous unique circRNAs may be produced from a single gene, resulting in a far greater number of circRNAs than protein-coding genes in human cells [20]. Recent studies have indicated that circRNAs, as important epigenetic regulators, could function by initiating and promoting endocrine resistance in breast cancer [21]. For instance, circRNA_0025202 was reported to inhibit tumor growth and tamoxifen resistance in breast cancer by sponging miR-182-5p and then upregulating FOXO3a expression [22]. circPVT1 was found to be highly expressed in ER^+^ breast cancer cells and tumor tissues, promoting ER^+^ breast tumorigenesis and endocrine resistance via both miRNA sponging and protein scaffolding effects [23]. Thus, gaining insight into the biological roles and regulatory mechanisms of functional circRNAs in response to endocrine therapy will help in overcoming resistance and further improve treatment outcomes.

circRNA-SFMBT2 (hsa_circ_0017639) is a circular RNA transcript originating from the host protein-coding gene SFMBT2. Recently, circRNA-SFMBT2 was reported to function as a tumor promoter in gastric cancer [24], non-small lung cancer [25, 26], and acute myeloid leukemia [27]. However, little is known about its regulatory role in other tumor types. Additionally, the mechanisms of circRNA-SFMBT2 reported in these papers were focused mostly on its role as a miRNA sponge. In addition to this well-known role, circRNAs may also perform their functions by interacting with proteins [28]. Considering that circRNA dysregulation is responsible for the emergence of cancer drug resistance [29], a deeper understanding of whether circRNA-SFMBT2 mediates endocrine resistance development via interactions with transcription factors to manipulate intracellular signaling pathways is needed. In this study, we investigated the regulatory role of circRNA-SFMBT2 in ERα signaling and examined its functional significance in cell growth and tamoxifen resistance in breast cancer.

## Results

### Characteristics of circRNA-SFMBT2 in breast cancer

**Fig. 1:**
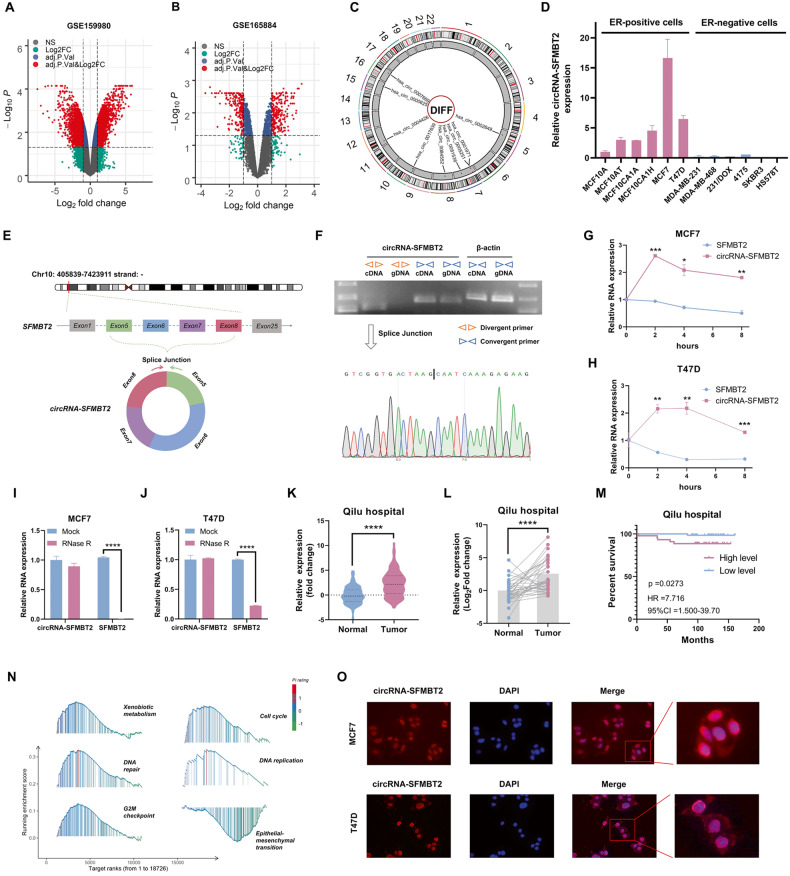
circRNA expression profiles in breast cancer and characterization of circRNA-SFMBT2. **A**, **B** Volcano plots showing differentially expressed circRNAs in GSE159980 (**A**) and GSE165884 (**B**). **C** Circos plot showing an overview of the genomic distribution of the 9 circRNA candidates. **D** qPCR analysis of circRNA-SFMBT2 expression in breast cancer cell lines. **E** Schematic representation of circRNA-SFMBT2. **F** PCR with convergent and divergent primers followed by Sanger sequencing was used to identify the loop structure of circRNA-SFMBT2. **G**, **H** qPCR analysis of the RNA levels of circRNA-SFMBT2 and SFMBT2 after treatment with actinomycin D (2 μg/ml). **I**, **J** qPCR analysis of the RNA levels of circRNA-SFMBT2 and SFMBT2 after treatment with RNase R. **K** qPCR analysis showing the expression levels of circRNA-SFMBT2 in 108 ER^+^ breast cancer tissues and 92 normal breast tissues. **L** qPCR analysis showing the expression levels of circRNA-SFMBT2 in 34 paired ER^+^ breast cancer tissues and adjacent normal tissues. **M** Kaplan–Meier survival analysis of patients with ER^+^ breast cancer with low and high circRNA-SFMBT2 expression. **N** GSEA of 16 breast cancer samples from the MiOncoCirc database. **O** RNA FISH analysis of circRNA-SFMBT2 in MCF7 and T47D cells. Experiments were conducted three times. In **G**–**K**, the data are shown as the means ± SDs and were analyzed using unpaired two-tailed Student’s *t*-test. The data in **L** were assessed by paired two-tailed *t*-test. Not significant (ns); *p < 0.05; **p < 0.01; ***p < 0.001; ****p < 0.0001.

We reanalyzed two public datasets from GEO to investigate circRNA expression profiles in breast cancer tissues as well as tamoxifen-resistant cells (Fig. 1A and B). Our analysis revealed that the expression of 9 circRNAs was upregulated in both breast cancer tissues and tamoxifen-resistant cells (Fig. S1A). A circos plot was used to visualize the overview of the genomic distribution of the 9 circRNA candidates (Fig. 1C). Subsequent qPCR analysis confirmed that circRNA-SFMBT2 had higher expression in ER^+^ breast cancer cells than in ER-negative (ER^_^) cells (Fig. 1D). In addition, circRNA-SFMBT2 expression appeared to increase with increasing malignancy from MCF10A normal breast epithelial cells to their premalignant derivative cell line (MCF10AT) and its malignant derivative cell lines (MCF10A1A and MCF10A1H). Based on these observations, we ultimately selected circRNA-SFMBT2 as an optimal target for further studies.

circRNA-SFMBT2 (circBase ID: hsa_circ_0017639) is generated from exons 5–8 of the SFMBT2 gene and is 536 nt in length (Fig. 1E). To examine whether circRNA-SFMBT2 is an endogenous circRNA in human cells, we performed polymerase chain reaction (PCR) analysis on in complementary DNA (cDNA) and genomic DNA (gDNA) using a convergent/divergent primer amplification strategy (Fig. 1F). Our results showed that circRNA-SFMBT2 could be amplified from cDNA but not from gDNA using the divergent primers. Subsequently, a backsplice junction site of exon 8 with exon 5 was identified by Sanger sequencing in the product amplified with the divergent primers for circRNA-SFMBT2. Next, the results of an RNA decay assay following transcriptional inhibition with actinomycin D demonstrated that the half-life of circRNA-SFMBT2 was much longer than that of its counterpart mRNA SFMBT2 (Fig. 1G, H). In addition, the results of an RNase R digestion assay confirmed that circRNA-SFMBT2 possessed a covalently closed loop structure that was resistant to digestion by RNase R (Fig. 1I, J).

### Upregulation of circRNA-SFMBT2 predicted poor prognosis and was associated with cell growth and the tamoxifen response in breast cancer

To elucidate the biological role of circRNA-SFMBT2, we measured the expression level of circRNA-SFMBT2 in 92 nontumor tissues and 108 tumor tissues from patients with ER^+^ breast cancer recruited from Qilu Hospital. qPCR analysis indicated that circRNA-SFMBT2 displayed much higher expression levels in ER^+^ breast cancer tissues than in normal breast tissues (Fig. 1K). Importantly, most breast cancer tissues exhibited higher expression of circRNA-SFMBT2 than the paired nontumor tissues (Fig. 1L). Subsequent survival analysis suggested that patients with high circRNA-SFMBT2 expression had shorter survival times than those with low circRNA-SFMBT2 expression (Fig. 1M). We proceeded to assess the association between the expression of circRNA-SFMBT2 and clinicopathological variables by categorizing all patients with ER^+^ breast cancer into high- and low-expression groups based on the average value of circRNA-SFMBT2 expression determined by PCR as the cutoff (Table 1). Our analysis revealed that high circRNA-SFMBT2 expression was significantly associated with larger tumor size, suggesting that circRNA-SFMBT2 may play a major role in breast cancer cell proliferation.

**Table 1:** Associations between circRNA-SFMBT2 expression and clinicopathological features in patients with ER+ breast cancer.

Features	Cases (*n* = 108)	circRNA-SFMBT2		*p*-value
		Low	High	
*Age*				
≤50	59	30	29	
>50	49	24	25	0.9814
*Histologic grade*				
G1	4	2	3	
G2	72	36	36	
G3	27	14	13	
Unknown	5	2	3	0.9997
*Tumor size*				
≤2	50	32	18	
>2	56	22	34	
Unknown	2	2	NA	0.0396^a^
*Lymph node metastasis*				
No	57	27	30	
Yes	49	26	23	
Unknown	2	1	1	0.9870
*Distant metastasis*				
No	82	41	41	
Yes	13	7	6	
Unknown	13	6	7	0.9971
*PR status*				
Neg	15	6	9	
Pos	93	48	45	0.7058
*HER2 status*				
Neg	61	32	29	
Pos	47	22	25	0.8440

^a^Significant.

Next, we used the MiOncoCirc database [30] to estimate the biological functions of circRNA-SFMBT2. Gene set enrichment analysis (GSEA) showed that xenobiotic metabolism and cell growth-related signaling pathways were highly enriched in circRNA-SFMBT2-high tumors (Fig. 1N), indicating that circRNA-SFMBT2 may participate in cell proliferation and drug response. In GSEA, the top-ranked genes can be used as phenotypic markers and thereby most likely represent the biological role of circRNA-SFMBT2 in breast cancer. Thus, we performed a single-sample gene set enrichment analysis (ssGSEA) with the top 50 genes to define an enrichment score that could reflect their degree of association with circRNA-SFMBT2, termed circRNA-SFMBT2-related signaling. Based on this signaling profile, we continued to investigate the effect of circRNA-SFMBT2 on the response to tamoxifen in patients with ER^+^ breast cancer from the TCGA and METABRIC databases via drug sensitivity prediction analysis using the oncoPredict R package [31]. Our analysis showed that patients with high circRNA-SFMBT2 expression were less sensitive to tamoxifen than those with low circRNA-SFMBT2 expression (Fig. S1B, C). Considering that the cellular distribution of a circRNA is responsible for how it exerts its biological roles, we utilized RNA FISH to examine the circRNA-SFMBT2 probe intensity in two ERα-positive cell lines, MCF7 and T47D. As shown in Fig. 1O, circRNA-SFMBT2 was widely distributed in cells, indicating that circRNA-SFMBT2 may play multiple roles in cellular signaling and cancer biology.

### QKI promoted circRNA-SFMBT2 biogenesis in breast cancer cells

**Fig. 2:**
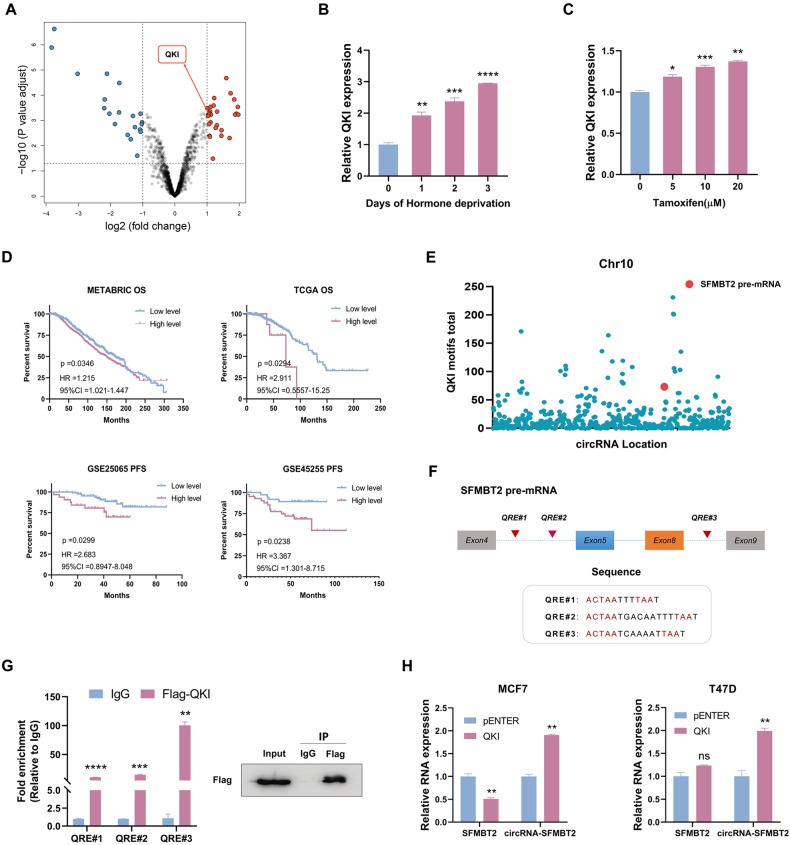
QKI promoted circRNA-SFMBT2 biogenesis in breast cancer cells. **A** The volcano plot shows differentially expressed mRNAs in tamoxifen-resistant breast cancer cells from GSE159980. **B** qPCR analysis showed that estrogen depletion increased the level of QKI mRNA in a time-dependent manner. **C** qPCR analysis showed that tamoxifen treatment increased the level of QKI mRNA in a dose-dependent manner. **D** Kaplan–Meier survival analysis of patients with ER^+^ breast cancer with low and high QKI expression. OS overall survival, PFS progression-free survival. **E** Distribution overview of QKI binding motifs on chromosome (Chr) 10. **F** A schematic showing three potential QREs in the introns flanking exon 5 and exon 8 of SFMBT2 pre-mRNA. **G** qPCR analysis of SFMBT2 pre-mRNA enriched by precipitation with an anti-Flag antibody or IgG in MCF7 cells expressing Flag-tagged QKI (left). The immunoprecipitation efficiency of Flag-QKI was evaluated by Western blotting (right). **H** qPCR analysis showing the expression levels of circRNA-SFMBT2 and SFMBT2 in breast cancer cells with or without QKI overexpression. The means ± SDs of three independent experiments were analyzed using unpaired two-tailed Student’s *t*-test unless otherwise noted. Not significant (ns), *p < 0.05, **p < 0.01, ***p < 0.001 and ****p < 0.0001 compared with the controls.

Some RNA-binding proteins, such as QKI, have been demonstrated to regulate circRNA biogenesis by binding to their flanking introns [17, 32]. In our analysis of data from GSE159980, we found that QKI expression was markedly elevated in tamoxifen-resistant cells (Fig. 2A). Moreover, the QKI expression level showed a significant gradient increase following hormone deprivation or tamoxifen treatment (Fig. 2B, C). Kaplan‒Meier survival analysis revealed that high QKI expression was associated with poor prognosis in patients with ER^+^ breast cancer in multiple datasets (Fig. 2D). Using ChIP-seq data from a previous study [17], we found dramatic enrichment of QKI binding motifs in the SFMBT2 pre-mRNA (Fig. 2E). As described in previous studies [17, 33, 34], QKI binding to circRNA flanking introns is dependent on the QKI response element (QRE), a bipartite consensus sequence (NACUAAY-N_1-20_-UAAY). By searching for the QRE sequence, we matched three putative QREs in the flanking introns of circRNA-SFMBT2 (Fig. 2F). Next, a RIP assay was performed to confirm that QKI could indeed bind the SFMBT2 pre-mRNA (Fig. 2G). Furthermore, we found that circRNA-SFMBT2 biogenesis could be driven by QKI overexpression (Fig. 2H; Fig. S1D, E), whereas the expression of the circRNA-SFMBT2 host gene SFMBT2 was largely unaltered or slightly reduced (Fig. 2H). Taken together, these results suggest that QKI may bind to the flanking introns upstream and downstream of the circRNA-forming exons in SFMBT2 pre-mRNA to facilitate circRNA-SFMBT2 biogenesis.

### circRNA-SFMBT2 enhanced cell growth and tamoxifen resistance in breast cancer

To explore the biological role of circRNA-SFMBT2 in breast cancer progression, we constructed circRNA-SFMBT2 overexpression vectors and designed two siRNAs specifically targeting the back-splicing region of circRNA-SFMBT2. The efficiencies of circRNA-SFMBT2 overexpression and knockdown were evaluated using qPCR analysis in both MCF7 and T47D cells (Fig. S2A, B). Regarding circRNA-SFMBT2 knockdown, si2-circRNA-SFMBT2 had a higher silencing efficiency than si1-circRNA-SFMBT2 and did not alter the expression of SFMBT2 in breast cancer cells. Hence, we selected si2-circRNA-SFMBT2 for use in subsequent experiments.

**Fig. 3:**
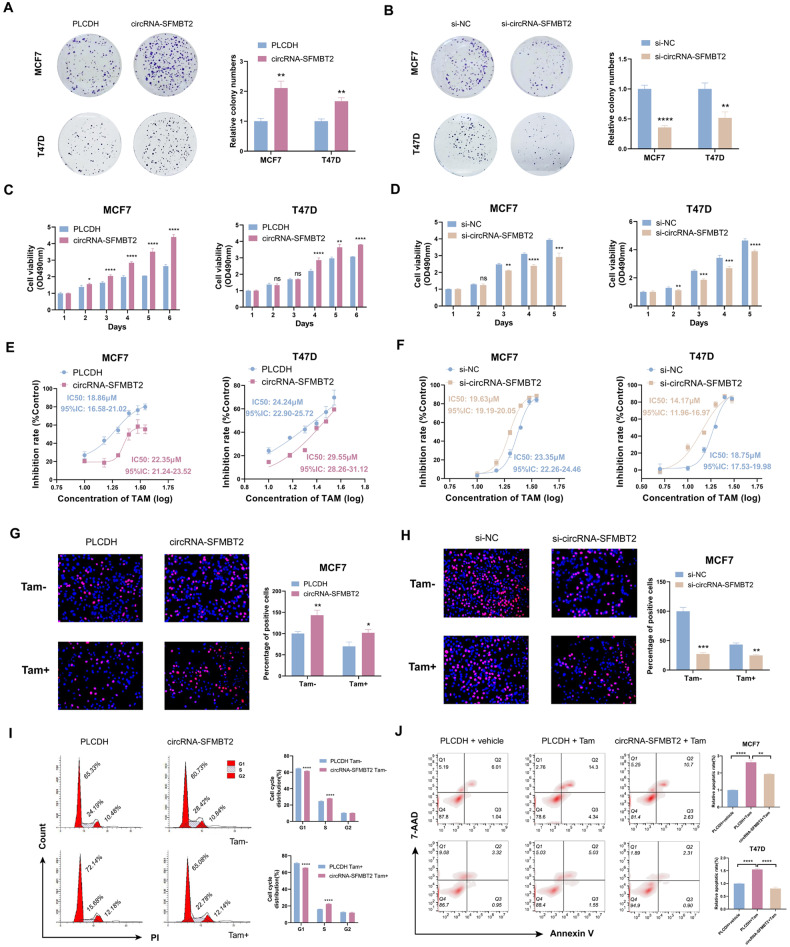
circRNA-SFMBT2 enhanced breast cancer cell proliferation and tamoxifen resistance. **A**–**D** Colony formation (**A**, **B**) and MTT (**C**, **D**) assays were used to evaluate the impact of circRNA-SFMBT2 on the proliferation of both MCF7 and T47D cells. **E**, **F** IC50 values measured by an MTT assay showing the effect of circRNA-SFMBT2 overexpression or silencing on tamoxifen sensitivity in both MCF7 and T47D cells. **G**, **H** Representative images of EdU incorporation by fluorescence microscopy. EdU incorporation assay showing the effect of circRNA-SFMBT2 overexpression or silencing on the growth of cells in the presence or absence of 5 μM tamoxifen (Tam). **I** The cell cycle distribution was analyzed using flow cytometry after transfected cells were treated with vehicle or 5 μM Tam for 48 h. **J** Apoptosis was analyzed by flow cytometry after transfected cells were treated with vehicle or 10 μM Tam for 48 h. The experiments were repeated three times, the data are presented as the means ± SDs and *p* values were calculated by unpaired two-tailed Student’s *t*-test unless otherwise noted. Not significant (ns); *p < 0.05; **p < 0.01; ***p < 0.001; ****p < 0.0001.

circRNA-SFMBT2 overexpression markedly enhanced but circRNA-SFMBT2 silencing effectively suppressed cell proliferation and tamoxifen resistance in vitro. These results were first demonstrated by colony formation (Fig. 3A, B) and MTT (Figs. 3C–F and S2D, E) assays in both MCF7 and T47D cells. Consistent with these findings, the results of the EdU incorporation assay demonstrated that overexpression of circRNA-SFMBT2 strikingly increased the percentage of EdU-positive MCF7 (Fig. 3G) and T47D (Fig. S2C) cells and that knockdown of circRNA-SFMBT2 greatly reduced the percentage of EdU-positive MCF7 cells (Fig. 3H). Furthermore, cell cycle analysis based on flow cytometry suggested that overexpressing circRNA-SFMBT2 accelerated the G1/S transition in MCF7 (Fig. 3I) and T47D (Fig. S2F) cells, whereas silencing circRNA-SFMBT2 blocked the G1/S transition to cause cell cycle arrest in MCF7 cells (Fig. S2G). Notably, these results did not change upon treatment with tamoxifen. Flow cytometric analysis of apoptosis indicated that overexpressing circRNA-SFMBT2 attenuated tamoxifen-induced apoptosis in both MCF7 and T47D cells (Fig. 3J). Collectively, our results demonstrate that circRNA-SFMBT2 is a necessary and sufficient factor for cell growth and tamoxifen resistance in breast cancer.

### circRNA-SFMBT2 facilitated ERα signaling in vitro and in vivo

**Fig. 4:**
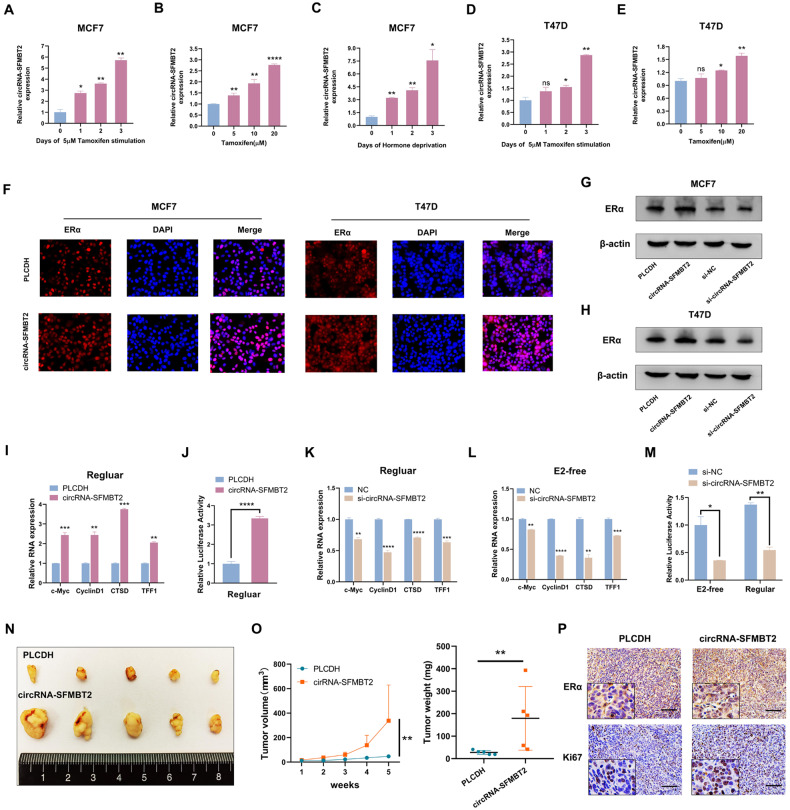
circRNA-SFMBT2 facilitated ERα signaling in vitro and in vivo. **A**–**E** Estrogen depletion or tamoxifen treatment promoted the expression of circRNA-SFMBT2 in both MCF7 and T47D cells. **F** IF staining with an anti-ERα antibody showing the effect of overexpressing circRNA-SFMBT2 on ERα protein levels in breast cancer cells. **G**, **H** Western blotting was used to evaluate the impact of circRNA-SFMBT2 overexpression or silencing on ERα protein levels in both MCF7 and T47D cells. **I** circRNA-SFMBT2 overexpression increased the levels of ERα target genes in MCF7 cells. **J** A luciferase reporter assay was used to evaluate the effect of circRNA-SFMBT2 overexpression on ERα transcriptional activity in 293T cells. **K**, **L** circRNA-SFMBT2 silencing reduced the levels of ERα target genes in MCF7 cells after 6 days of treatment with regular or E2-free medium. **M** A luciferase reporter assay was used to evaluate the effect of circRNA-SFMBT2 silencing on ERα transcriptional activity in 293T cells after 6 days of treatment with a regular or E2-free medium. **N** Images of xenograft tumors from each group. **O** The volume (left) and weight (right) of subcutaneous xenograft tumors. **P** Representative images of IHC staining for Ki67 and ERα in samples from the two groups. Scale bars = 100 μm. The differences in tumor volume and weight between the two groups were analyzed by the Mann‒Whitney test. Not significant (ns); *p < 0.05; **p < 0.01; ***p < 0.001; ****p < 0.0001.

After short-term exposure to tamoxifen, the levels of drug-resistant transcripts in breast cancer cells can become increased to attenuate the inhibitory effect of tamoxifen on ERα transcriptional activity [35–37]. To further examine the role of circRNA-SFMBT2 in the development of tamoxifen resistance, we treated breast cancer cells with tamoxifen or with estradiol (E2)-free medium. qPCR analysis showed that treatment with tamoxifen or E2-free medium dramatically increased the circRNA-SFMBT2 expression level in a time- or dose-dependent manner in MCF7 cells (Fig. 4A–C). Although T47D cells appeared to be less responsive than MCF7 cells to E2 inhibition, a similar trend was still observed following the same protocol of treatment with tamoxifen (Fig. 4D, E). We continued to investigate estrogen-mediated regulation of circRNA-SFMBT2 over a time course after E2 stimulation in breast cancer cells. After E2 stimulation, the expression of circRNA-SFMBT2 appeared to decrease transiently over a 24-h period and then increase significantly in both MCF7 and T47D cells (Fig. S3A, B). In summary, these observations strongly suggest that circRNA-SFMBT2 is an estrogen-regulated circRNA and may play an essential role in the development of tamoxifen resistance in breast cancer.

As alterations in components of ERα signaling are responsible for tamoxifen resistance, we further investigated the relationship between circRNA-SFMBT2 and ERα protein levels in both MCF7 and T47D cells. As shown in Fig. 4F, IF staining indicated that overexpressing circRNA-SFMBT2 greatly increased the ERα protein level. Consistent with this finding, Western blot analysis confirmed that circRNA-SFMBT2 overexpression could increase the level of the ERα protein, whereas ERα protein expression was significantly inhibited following circRNA-SFMBT2 silencing (Fig. 4G, H). To identify the regulatory role of circRNA-SFMBT2 in ERα transcriptional activity, we carried out qPCR analysis using primers specific for four widely used ERα target genes: c-Myc, CyclinD1, CTSD, and TFF1. Our results indicated that circRNA-SFMBT2 overexpression considerably promoted the transcription of ERα target genes (Fig. 4I). Moreover, circRNA-SFMBT2 enhanced ERE luciferase activity in a dual luciferase assay (Fig. 4J). Consistent with the data of circRNA-SFMBT2 overexpression, circRNA-SFMBT2 knockdown led to markedly decreased transcript levels of ERα target genes (Fig. 4K). In addition, circRNA-SFMBT2 knockdown significantly reduced ERE luciferase activity (Fig. 4M). Notably, we also observed a similar result in an E2-deprived environment (Fig. 4L, M). Subsequently, we proceeded to evaluate the roles of circRNA-SFMBT2 through in vivo experiments. Our data revealed that circRNA-SFMBT2 overexpression markedly accelerated tumor growth in vivo (Fig. 4N, O). Consistent with these findings, immunohistochemical (IHC) staining showed that ERα and Ki67 protein levels were significantly higher in tumor tissues with circRNA-SFMBT2 overexpression than in control group tissues (Fig. 4P).

### circRNA-SFMBT2 interacted with the ERα protein

**Fig. 5:**
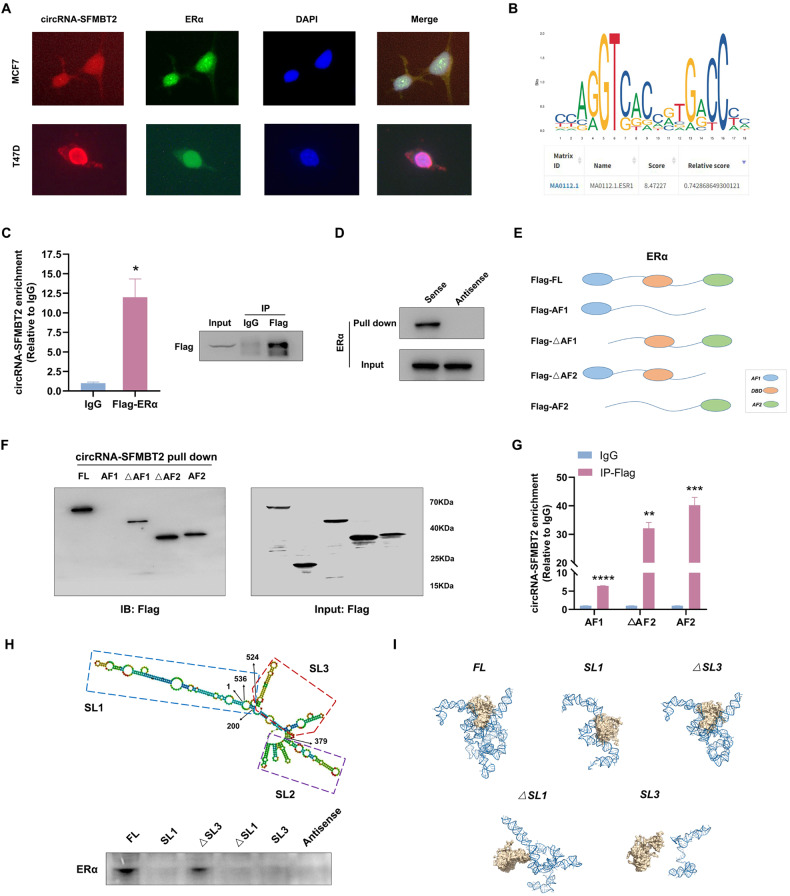
circRNA-SFMBT2 interacted with the ERα protein. **A** RNA FISH combined with protein IF showing the subcellular localization of circRNA-SFMBT2 and ERα in MCF7 and T47D cells. **B** Motif analysis using the JASPAR database. **C** qPCR analysis of circRNA-SFMBT2 enriched by precipitation with an anti-Flag antibody or IgG in MCF7 cells transfected with Flag-ERα (left). The immunoprecipitation efficiency of Flag-ERα was determined using Western blotting (right). **D** An RNA pulldown assay was used to detect endogenous ERα precipitated by the biotinylated sense and antisense probes of circRNA-SFMBT2 in MCF7 cells. The antisense probe was used as a negative control. **E** A schematic map of the ERα truncation mutants. **F** Western blot analysis of the circRNA-SFMBT2 binding capability in 293T cells. **G** qPCR analysis of circRNA-SFMBT2 enriched by precipitation with an anti-Flag antibody or IgG in 293T cells expressing Flag-tagged truncated ERα. **H** The secondary structure of circRNA-SFMBT2 was predicted using the RNAfold web server (upper). circRNA-SFMBT2 was divided into three stem‒loop regions. SL1: 525–200 nt; SL2: 201–379 nt; SL3: 380–524 nt. (lower) Western blotting of ERα pulled down by truncated circRNA-SFMBT2 in MCF7 cells. **I** Graphical representation of the molecular docking between ERα and the circRNA-SFMBT2 truncations using the HDOCK server. The data were based on the results of three independent experiments and are presented as the means ± SDs. *p < 0.05, **p < 0.01, and ***p < 0.001 compared with the controls.

Because noncoding RNAs often interact with some transcription factors to affect gene expression [38], we next investigated the crosstalk between circRNA-SFMBT2 and the ERα protein. RNA FISH combined with protein IF was performed to identify that circRNA-SFMBT2 was strongly colocalized with the ERα protein in both MCF7 and T47D cells (Fig. 5A). In addition, motif analysis using the JASPAR database confirmed that circRNA-SFMBT2 contained the highly conserved sequence for binding to the ERα protein (Fig. 5B). To test these findings, RIP was first performed to identify that circRNA-SFMBT2 could be precipitated with the anti-Flag antibody in MCF7 cells transfected with Flag-ERα (Fig. 5C). The RNA pulldown assay showed that the sense transcript of circRNA-SFMBT2 could indeed bind to endogenous ERα, but antisense transcript could not (Fig. 5D). As outlined in Fig. 5E, we constructed a series of ERα truncation mutants, including AF1, AF2, △AF1 (deletion of AF1), and △AF2 (deletion of AF2), to identify the exact domain of ERα that binds to circRNA-SFMBT2. RNA pulldown assays demonstrated that the three truncation mutants containing the AF2 and/or DBD domain retained efficient binding affinity for circRNA-SFMBT2, but the truncation mutant containing only the AF1 domain lost the ability to bind circRNA-SFMBT2 (Fig. 5F). A RIP assay was then performed to confirm that the AF2 and DBD domains indeed had greater binding affinity for circRNA-SFMBT2 than the AF1 domain (Fig. 5G). To map the ERα binding region within circRNA-SFMBT2, the RNAfold algorithm [39] was used to predict the secondary structure of circRNA-SFMBT2 with the minimum free energy (Fig. 5H, upper panel). Based on the RNAfold prediction, we attempted to define three different stem-loop regions, named stem-loop 1 (SL1), stem-loop 2 (SL2), and stem-loop 3 (SL3) and then designed four deletion mutants of circRNA-SFMBT2. The subsequent RNA pulldown assay showed that the SL3 deletion mutant (△SL3) consisting of only SL1 and SL2 could pull down endogenous ERα as efficiently as full-length circRNA-SFMBT2, but intriguingly, we found no evidence that SL1 or SL2 in the other mutants had the capability to bind to ERα (Fig. 5H, lower panel). Hence, we hypothesized that the tertiary structure formed by SL1 and SL2 might play an important role in the process of ERα binding. To further test our hypothesis, the HDOCK server [40] was used to assess the interaction between ERα and distinct deletion mutants of circRNA-SFMBT2. Consistent with the results from the RNA pulldown assay, the results of docking analysis (Fig. 5I) indicated that the tertiary structure of circRNA-SFMBT2 had a high binding affinity for ERα, and deleting only SL3 did not abolish the capability of circRNA-SFMBT2 to bind to ERα.

### circRNA-SFMBT2 inhibited ERα degradation via the ubiquitin–proteasome pathway

**Fig. 6:**
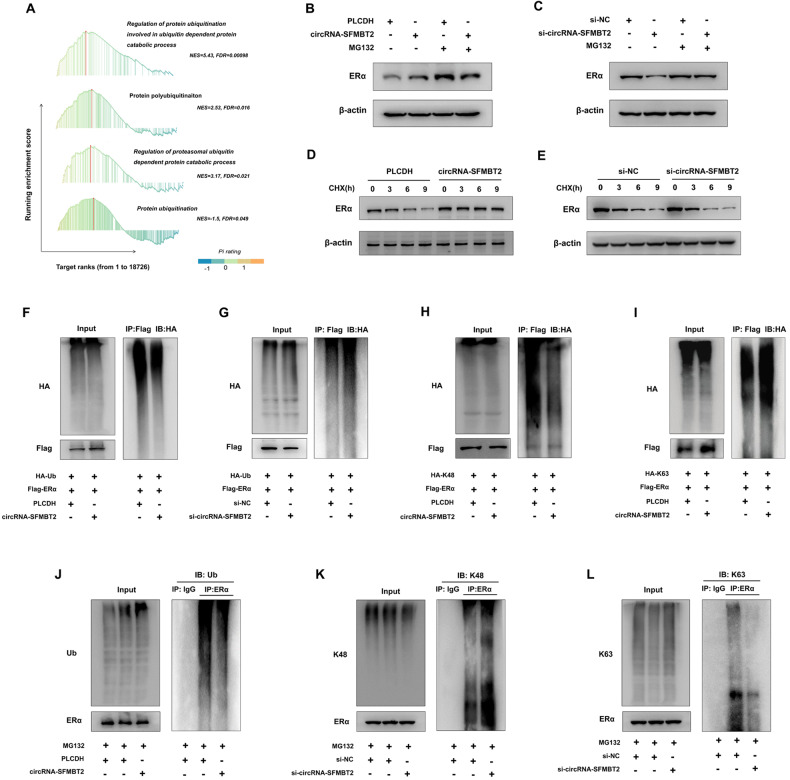
circRNA-SFMBT2 inhibited ERα degradation via the ubiquitin–proteasome pathway. **A** GSEA showing enrichment of ubiquitination-related signaling in samples with high levels of circRNA-SFMBT2. **B**, **C** Western blot analysis showing the impact of circRNA-SFMBT2 overexpression (**B**) or silencing (**C**) on ERα protein expression in MCF7 cells after treatment with 10 μM MG132 for 6 h. **D**, **E** Western blot analysis showing the impact of circRNA-SFMBT2 overexpression (**D**) or silencing (**E**) on ERα protein expression in MCF7 cells after treatment with 20 μg/ml CHX for the indicated times. **F**, **G** Ubiquitinated ERα was detected by immunoprecipitation with an anti-Flag antibody and immunoblotting with an anti-HA antibody in 293 T cells with circRNA-SFMBT2 overexpression (**F**) or silencing (**G**) and control cells. **H**, **I** Western blot analysis indicated that circRNA-SFMBT2 overexpression decreased K48-linked ubiquitination of ERα (**H**) but increased K63-linked ubiquitination of ERα (**I**) in 293 T cells. **J** circRNA-SFMBT2 overexpression decreased endogenous ubiquitination of ERα in MCF7 cells. **K**, **L** circRNA-SFMBT2 silencing promoted endogenous K48-linked ubiquitination of ERα but suppressed endogenous K63-linked ubiquitination of ERα in MCF7 cells.

To explore the mechanism by which circRNA-SFMBT2 affected the ERα protein level, we performed GSEA using the MiOncoCirc database to identify significantly enriched biological processes associated with these circRNA-SFMBT2-regulated genes. Our analysis showed that protein ubiquitination-related signaling was markedly activated in the high circRNA-SFMBT2 expression group (Fig. 6A). The ubiquitin–proteasome pathway is a key pathway for controlling protein degradation and recycling in most cellular processes [41]. Therefore, we speculated that circRNA-SFMBT2 may abolish ubiquitination-mediated ERα degradation by interacting with the ERα protein. Indeed, the addition of MG132 reversed circRNA-SFMBT2 overexpression-mediated ERα enrichment (Fig. 6B) and circRNA-SFMBT2 knockdown-mediated ERα degradation (Fig. 6C). The results of the cycloheximide (CHX) chase assay revealed that forced expression of circRNA-SFMBT2 significantly prolonged the half-life of ERα protein (Fig. 6D), whereas the ERα protein half-life was markedly shortened by circRNA-SFMBT2 silencing (Fig. 6E). We then performed a ubiquitination-based immunoprecipitation assay and found that circRNA-SFMBT2 overexpression significantly diminished the amount of ubiquitinated ERα (Fig. 6F), which was markedly increased upon circRNA-SFMBT2 silencing (Fig. 6G). Moreover, we further demonstrated that circRNA-SFMBT2 significantly inhibited K48-linked ubiquitination of ERα (Fig. 6H) but promoted its K63-linked ubiquitination (Fig. 6I). Consistent with the results of the Flag-ERα pulldown assay in 293 T cells, our data showed that endogenous ERα ubiquitination was significantly reduced in MCF7 cells overexpressing circRNA-SFMBT2 (Fig. 6J). Furthermore, knocking down circRNA-SFMBT2 in MCF7 cells markedly enhanced the K48-linked ubiquitination of endogenous ERα (Fig. 6K) while decreasing the K63-linked ubiquitination of endogenous ERα (Fig. 6L).

### circRNA-SFMBT2 stabilized ERα in an RNF181-dependent manner

**Fig. 7:**
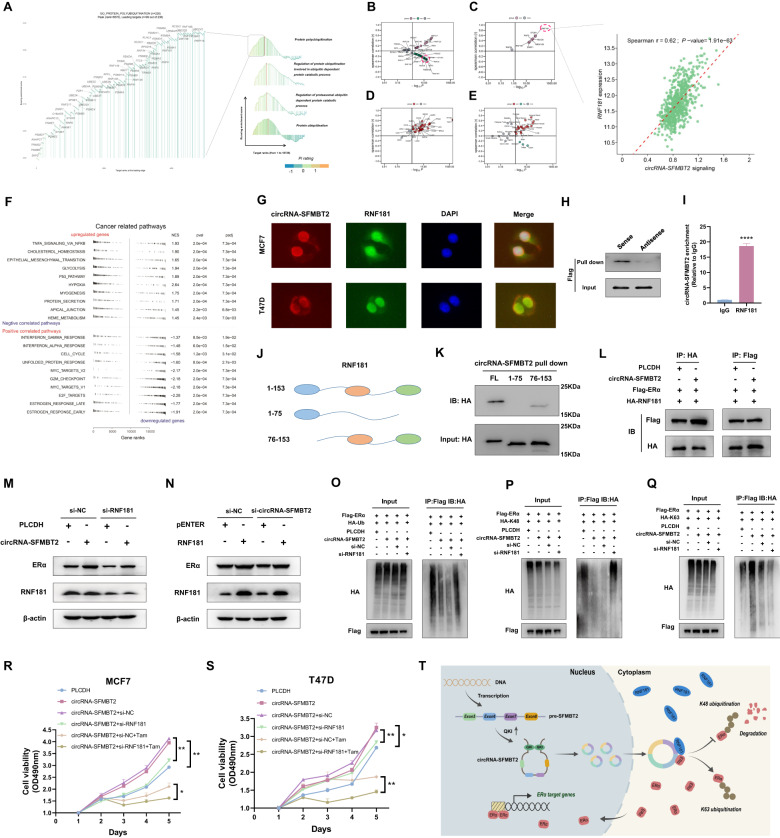
circRNA-SFMBT2 stabilized ERα in an RNF181-dependent manner. **A** Rank-based GSEA of genes significantly modulated by circRNA-SFMBT2 identified polyubiquitination-related genes that were preferentially promoted or suppressed by circRNA-SFMBT2. **B**–**E** Spearman correlation analysis. The correlation of ubiquitination-related genes with tamoxifen sensitivity in TCGA ER^+^ breast cancer cohorts (**B**). RNF181 exhibited the strongest association with circRNA-SFMBT2 signaling in ER^+^ breast cancer cohorts from TCGA (**C**). The correlation of circRNA-SFMBT2 signaling with RNF181 in the TCGA (**D**) and GTEx (**E**) pancancer datasets. **F** GSVA showing cancer-related pathways strongly correlated with RNF181 in the data from GSE143947. **G** RNA FISH combined with protein IF showing the subcellular localization of circRNA-SFMBT2 and RNF181 in MCF7 and T47D cells. **H** Western blot analysis showing Flag-tagged RNF181 retrieved by biotinylated sense and antisense probes of circRNA-SFMBT2 in 293T cells transfected with Flag-RNF181. The antisense probe was used as the negative control. **I** qPCR analysis of circRNA-SFMBT2 enriched by precipitation with an anti-RNF181 antibody or IgG in MCF7 cells. **J** A schematic map of the RNF181 truncation mutants. **K** Western blot analysis of HA-tagged full-length and truncated RNF181 pulled down by in vitro-transcribed circRNA-SFMBT2 in 293T cells. **L** Overexpressing circRNA-SFMBT2 enhanced the interaction of RNF181 with ERα. **M** Silencing RNF181 abrogated the circRNA-SFMBT2-mediated stabilization of ERα. **N** circRNA-SFMBT2 knockdown decreased the RNF181-mediated stabilization of ERα. **O** Silencing RNF181 reversed the suppression of ERα ubiquitination induced by circRNA-SFMBT2 overexpression. **P**, **Q** Silencing RNF181 reversed the impact of circRNA-SFMBT2 overexpression on K48-linked (**P**) and K63-linked (**Q**) ubiquitination of ERα. **R**, **S** Silencing RNF181 abrogated the promotion of cell growth and tamoxifen resistance by overexpression of circRNA-SFMBT2 in MCF7 (**R**) and T47D (**S**) cells. **T** A schematic diagram showing that circRNA-SFMBT2 overexpression could drive ERα signaling. The means ± SDs of three independent experiments were analyzed using unpaired two-tailed Student’s *t*-test unless otherwise noted. *p < 0.05, **p < 0.01, ****p < 0.0001 compared with the controls.

To determine the potential ubiquitin-protein E3 ligase responsible for circRNA-SFMBT2-mediated ERα stabilization, we extracted the top-ranked genes modulated by circRNA-SFMBT2 from the protein polyubiquitination gene set according to the GSEA enrichment score (Fig. 7A). Following drug response prediction with oncoPredict, Spearman correlation analysis was performed to identify the ubiquitination-related genes with a highly negative correlation to tamoxifen sensitivity in TCGA ER^+^ breast cancer cohorts (Fig. 7B). Among these genes, RNF181 had the strongest correlation with circRNA-SFMBT2 signaling in the TCGA dataset (Fig. 7C). Concordantly, Spearman correlation analysis using the data from the TCGA and GTEx projects revealed that RNF181 expression had a significant positive correlation with circRNA-SFMBT2 signaling in most cancer types, particularly in the TCGA pancancer dataset (Fig. 7D, E). Subsequent gene set variation analysis (GSVA) showed that RNF181 expression exhibited the strongest positive correlation with ERα-mediated signaling (Fig. 7F). RNF181 is an E3 ligase that has been reported to participate in the regulation of ERα protein stability [12]. In this work, we reassessed the interaction of RNF181 with ERα by immunoprecipitation analysis and found that ERα could bind to RNF181 in a manner dependent on its AF1 domain (Fig. S4A). Thus, we proceeded to investigate whether RNF181 can use circRNA-SFMBT2 as a scaffold for its efficient regulation of ERα protein ubiquitination.

The data from RNA FISH combined with protein IF showed that circRNA-SFMBT2 was colocalized with RNF181 in both MCF7 and T47D cells, indicating the possibility of binding between circRNA-SFMBT2 and RNF181 (Fig. 7G). RNA pulldown followed by Western blot analysis demonstrated that circRNA-SFMBT2 could interact with the RNF181 protein (Fig. 7H). RIP followed by qPCR analysis confirmed that circRNA-SFMBT2 was markedly precipitated by the anti-RNF181 antibody (Fig. 7I). To detect the RNF181 domain that interacts with circRNA-SFMBT2, we constructed HA-tagged wild-type RNF181 and a panel of deletion mutants of RNF181, as shown in Fig. 7J. The subsequent RNA pulldown assay indicated that amino acids 76–153, containing a RING domain, were required for circRNA-SFMBT2 binding (Fig. 7K), whereas amino acids 1–75 have been reported to be involved in ERα binding. These data showed that circRNA-SFMBT2 did not interfere with the interaction between RNF181 and ERα. Hence, circRNA-SFMBT2 can serve as a scaffold to recruit RNF181 to ERα to reinforce ERα stability.

Next, the results of two different immunoprecipitation assays demonstrated that circRNA-SFMBT2 overexpression could markedly reinforce the interaction between RNF181 and ERα (Fig. 7L). Additionally, we found that overexpressing circRNA-SFMBT2 reversed RNF181 silencing-mediated ERα degradation (Fig. 7M), while overexpressing RNF181 dampened circRNA-SFMBT2 silencing-mediated ERα degradation (Fig. 7N). To further confirm whether RNF181 can regulate the effects of circRNA-SFMBT2 on ERα ubiquitination, we performed in vitro ubiquitination assays. Our results suggested that RNF181 knockdown strongly impaired the effect of circRNA-SFMBT2 on ERα ubiquitination (Fig. 7O–Q). Moreover, we further discovered that RNF181 silencing abrogated circRNA-SFMBT2-induced cell growth and tamoxifen resistance (Fig. 7R, S).

## Discussion

Breast cancer is the most common malignant tumor threatening the health of women worldwide [42]. Approximately 70% of patients with breast cancer have ERα expression; thus, ERα is an important therapeutic target in breast cancer. However, the emergence of resistance drastically limits the clinical benefits of endocrine therapy and poses a considerable challenge to basic and clinical research [43]. In this study, we elucidated a previously unrecognized role for circRNA-SFMBT2 in regulating tumor progression and drug resistance (Fig. 7T), revealing a potential therapeutic target to improve efficacy and overcome resistance in tamoxifen-based treatment of breast cancer.

circRNAs are a novel class of noncoding RNAs that are generated via back-splicing and are involved in many cellular functions [44]. Accumulating evidence suggests that dysregulation of circRNAs contributes to the development and progression of breast cancer [45]. In this work, we found that circRNA-SFMBT2 expression exhibited a significant increase not only in primary breast cancer tissues but also in tamoxifen-resistant cells. In addition, circRNA-SFMBT2 was highly expressed in ER^+^ breast cancer cells in comparison to ER^-^ cells. Clinically, high expression levels of circRNA-SFMBT2 were associated with larger tumor size and poor prognosis in patients with ER^+^ breast cancer. Moreover, our findings showed that circRNA-SFMBT2 upregulation can be driven via the binding of the QKI protein to QRE sequences flanking the circRNA-SFMBT2-forming exons. The QKI protein, which plays a dual functional role as a tumor promoter and tumor suppressor, has been reported in some studies [46–48]. Here, our observations indicated that QKI expression could be induced by stimulation with tamoxifen or estrogen deprivation, and thus, its downstream effect is likely to be involved in the modulation of tamoxifen sensitivity in breast cancer cells. As expected, tamoxifen treatment of breast cancer cells indeed resulted in a significant increase in circRNA-SFMBT2 expression. Since tamoxifen can inhibit ERα signaling by antagonizing the binding of estrogen to ERα, the positive feedback loop between circRNA-SFMBT2 and ERα probably serves as a fail-safe mechanism to reverse the suppression of ERα signaling in response to tamoxifen treatment. Indeed, we observed that overexpressing circRNA-SFMBT2 not only promoted estrogen-dependent cell growth but also conferred estrogen-independent cell growth and tamoxifen resistance. Considering that aberrations in ERα signaling are closely associated with endocrine resistance, we proceeded to investigate the interplay between circRNA-SFMBT2 and ERα.

Previous studies revealed that ERα is still expressed in the majority of cases with endocrine resistance, implying that drug resistance development in these cases is not due to the loss of the ERα protein [5]. Clinically, ESR1 mutations have been identified as important drivers of resistance to aromatase inhibitors, but these mutations are not strongly linked to tamoxifen resistance [49]. To this end, we investigated whether circRNA-SFMBT2 is responsible for ERα protein stability during the development of tamoxifen resistance. Our results showed that overexpressing circRNA-SFMBT2 markedly increased the ERα protein level both in vitro and in vivo. Indeed, significant increases in ERα activity and the expression of its target genes were observed in breast cancer cells overexpressing circRNA-SFMBT2. Conversely, circRNA-SFMBT2 silencing markedly repressed ERα transcriptional activity. In addition, our data suggested that circRNA-SFMBT2 could simultaneously modulate estrogen-dependent and estrogen-independent gene expression. These results were further supported by the mode of circRNA-SFMBT2 binding to ERα, in which circRNA-SFMBT2 can interact with the AF2 and DBD domains of ERα. As a general rule, AF2 domain binding is ligand-dependent, whereas DBD domain binding is ligand-independent [8]. Activation of the ligand-independent domain can promote tumor escape from estrogen dependence to facilitate the development of endocrine resistance. In addition, we identified a region for tight binding with ERα in circRNA-SFMBT2. However, further studies are needed to better understand the detailed binding interaction between circRNA-SFMBT2 and ERα at the molecular level.

Ubiquitination is an essential posttranslational modification involved in many cellular processes that occur in normal physiological and disease states [50]. Our results indicated that circRNA-SFMBT2 was significantly associated with the ubiquitin–proteasome pathway in breast cancer. Remarkably, circRNA-SFMBT2 can control nonproteolytic ubiquitination to stabilize the ERα protein in ERα-positive cells, supporting a role for circRNA-SFMBT2 in the regulation of ERα signaling. Furthermore, our studies revealed that circRNA-SFMBT2 could serve as a scaffold to recruit RNF181 to regulate the ERα protein level in ERα-positive cells. This RNF181/circRNA-SFMBT2/ERα ternary complex reduced ubiquitination-mediated ERα degradation and activated ERα signaling to facilitate cell growth and tamoxifen resistance in breast cancer. Although the RNF181-ERα interaction has been described in the previous literature [12], our study is the first to reveal that RNF181 exerts its ubiquitin-ligase activity in a manner dependent on its RNA-binding capability, and circRNA-SFMBT2 was required for efficient ERα ubiquitination regulated by RNF181. Collectively, these findings revealed a previously unrecognized mechanism of ERα ubiquitination and thus provided novel insight into the role of circRNA-SFMBT2 in the regulation of protein metabolism.

In conclusion, our study demonstrated that circRNA-SFMBT2 could orchestrate ERα activation and render breast cancer cells resistant to tamoxifen. These observations suggested that antagonizing circRNA-SFMBT2 expression may serve as an alternative or complementary strategy to overcome tamoxifen resistance as well as to inhibit breast cancer progression.

## Materials and methods

### Data collection

GSE159980 and GSE165884 from Gene Expression Omnibus (GEO) were collected to investigate circRNA expression profiles in breast cancer tissues and tamoxifen-resistant cells. Breast cancer samples from MiOncoCirc database were enrolled to analyze the biological function of circRNA-SFMBT2. Tissue samples with follow-up data were recruited from ER^+^ breast cancer patients diagnosed and treated at Qilu Hospital of Shandong University. Written informed content was obtained from each patient before study participation. This study was approved by the Ethics Committee on Scientific Research of Shandong University.

### Cell culture and treatments

All cell lines used for this study were purchased from American Type Culture Collection (ATCC) and authenticated by short tandem repeats (STR) analysis. MCF7 and HEK-293T (293T) cells were cultured in high-glucose DMEM (Macgene, Beijing, China), and T47D cells were cultured in RPMI-1640 (Macgene, Beijing, China). Cell culture medium was supplemented with 10%FBS (HyClone, UT, USA), 100 U/ml penicillin (Macgene, Beijing, China), and 100 μg/ml streptomycin (Macgene, Beijing, China). All the cells were cultured in a humidified incubator with 37 °C and 5% CO_2_. Actinomycin D (Sigma-Aldrich, MO, USA) or cycloheximide (Selleck, TX, USA) treated cells to inhibit RNA or protein synthesis, respectively. MG132 (Selleck, TX, USA) was used to inhibit proteasome degradation.

### Plasmids construction and transfection

circRNA-SFMBT2 overexpressing plasmid pLCDH-circRNA-SFMBT2 and control plasmid pLCDH-ciR were purchased from GenePharma (Shanghai, China). The pENTER-C-Flag vector was used for the construction of a full-length QKI overexpressing vector. Coding sequences of full-length ERα and its truncated proteins were cloned into pFLAG-CMV-2 vectors (Sigma, MO, USA) expressed a fused protein with an N-terminal Flag. The pENTER-C-Flag vector containing full-length RNF181 was sourced from Vigene Biosciences (Rockville, MD, USA). Coding sequences of HA-tagged full-length and truncated RNF181 were constructed into pcDNA3.1 expression vectors. Three EREs (3 × ERE) were cloned into a pGL3-basic luciferase vector to construct a pGL3-(ERE)_3_ vector. Plasmid transfection was carried out using lipofectamine 2000 (Invitrogen, CA, USA). MCF7 cells stably overexpressing circRNA-SFMBT2 and control cell lines were established by puromycin selection for at least 4 weeks.

### Cell viability and cytotoxicity assays

MTT assay was used to detect cell viability and cytotoxicity. Briefly, transfected cells were used to seed 96-well plates at a density of 1 × 10^3^ or 2 × 10^3^ cells/well for cell viability or IC_50_ values measure, respectively. For the IC_50_ assay, the media was replaced with fresh media with a range of drug concentrations after cells were attached to the plate and continued to incubate for 48–72 h. Then, 20 μl of MTT (5 mg/ml, Sigma) was added to each well. After 4–6 h incubation, the supernatants were discarded, and then 100 μl/well of DMSO was added to dissolve the resulting formazan product. Finally, the absorbance at 490 nm was quantified using a microplate reader (Bio-Rad, CA, USA).

Colony formation and EdU incorporation assays were used to assess cell viability. For colony formation assay, cells with indicated treatment were seeded into a six-well plate at a density of 800 cells/well, and then incubated for another 14 days. 14 days later, cells were washed with PBS twice, fixed with methanol for 15 min, and stained with 0.2% crystal violet for 20 min. For the EdU incorporation assay, 50 μM EdU was added to each well containing treated cells in a 96-well plate. After incubation for 2 h, a subsequent procedure was performed according to the protocol described in the kit (RiboBio, China). EdU-stained fluorescent images were acquired with a fluorescence microscope (Carl Zeiss, Germany).

### Flow cytometry

Following indicated treatments, cells were harvested with trypsin and rinsed twice with PBS. After staining for 30 min with propidium iodide (Beyotime, Shanghai, China), cell cycle distribution was detected by a flow cytometer (BD Biosciences, NJ, USA). Cell apoptosis assay was performed using the BD PE Annexin V Apoptosis Detection Kit (BD Biosciences, NJ, USA), and analyzed by flow cytometry.

### Quantitative real-time PCR (qPCR)

Total RNAs from cells were isolated using trizol (Vazyme, Nanjing, China), and the RNA purity and concentration were assessed via a NanoDrop 2000 (Thermo Fisher Scientific, Waltham, USA). 0.5 μg of RNA was reverse transcribed into cDNA using Takara reverse transcription Kit (Shiga, Japan), and then qPCR was performed to quantify the RNA levels using the SYBR Green PCR mix (Takara). β-actin was used as the reference gene. Primer sequences used for this work were provided in Table S1.

### Dual-luciferase reporter assay

At 48 h after transfection with PRL-TK and pGL3-(ERE)_3_ plasmids, luciferase assay was performed using a dual luciferase reporter assay kit (Promega, WI, USA). The ratio of firefly to Renilla luciferase activity was used to define the final luminescence values.

### Immunofluorescence (IF) assay

Cells were plated onto glass coverslips in a 24-well plate. The following day, the cells were washed thrice with PBS, fixed with 4% paraformaldehyde (PFA) for 15 min, permeabilized with 0.3% TritonX-100 for 25 min, blocked with 10% goat serum for 1 h, and incubated with primary antibodies overnight at 4 °C. The next day, the cells were stained with secondary antibodies for 1 h, and fluorescent images were obtained using a fluorescence microscope (Leica, Wetzlar, Germany).

### RNA fluorescence in situ hybridization (FISH) assay

A Cy3-labeled probe targeting the splicing junction of circRNA-SFMBT2 was designed to perform a FISH assay, which procedure was conducted using the protocol provided by the RNA FISH Kit (GenePharma, Jiangsu, China). The location of circRNA-SFMBT2 in cells was observed, and images were acquired on a fluorescence microscope (Leica, Wetzlar, Germany). The sequence of the FISH probe was listed in Table S2.

### Western blotting

Protein samples were separated by SDS–PAGE gel electrophoresis and transferred onto a polyvinyl difluoride (PVDF) member. After blocking with 5% non-fat milk for 1 h, membranes were incubated with primary antibodies overnight at 4 °C. Followed by an incubation with anti-mouse or rabbit secondary antibodies, protein bands were visualized using an ECL detection kit (Vazyme, Nanjing, China). The antibodies used in the study were listed in Table S3.

### Co-immunoprecipitation (Co-IP)

To assess protein–protein interactions, cells were lysed with a lysis buffer for Western and IP (Beyotime, Jiangsu, China) supplemented with protease inhibitors. The supernatant of cell lysates was collected by a centrifuge at 12,000 rpm for 30 min at 4 °C, and subsequently precleared using Protein A/G Agarose beads. After incubation with the corresponding primary antibody for 2 h at 4 °C, the supernatant was used for immunoprecipitation by the addition of beads with rotation overnight at 4 °C. The following day, beads were washed five times with the NP-40 Lysis Buffer (Beyotime), and then bound proteins were eluted from the beads by boiling in an SDS loading buffer. Afterward, the immunoprecipitates were analyzed using Western blotting.

### RNA immunoprecipitation (RIP) assay

RIP assay was carried out using a Magna RIP RNA-Binding Protein Immunoprecipitation Kit (Millipore, MA, USA). Briefly, approximately 1 × 10^7^ cells were lysed with RIP lysis buffer supplemented with protease inhibitor cocktail and RNase inhibitor. Specific antibodies were added to 50 μl (per immunoprecipitation) of magnetic beads and incubated with rotation for 30 min at room temperature. Next, the cell lysates were thawed quickly and precipitated by centrifuge at 14,000 rpm for 10 min at 4 °C. The collected supernatant was used to perform RNA immunoprecipitation by incubation with antibody-coated beads overnight at 4 °C. After purification of the RNAs pulled down, qPCR was performed to analyze the level of circRNA-SFMBT2 enrichment in each group.

### RNA pulldown assay

To detect the RNA–protein interactions, RNA pulldown assay was performed using Pierce Magnetic RNA–Protein Pull-Down Kit (Thermo Fisher, MA, USA) according to manufacturer instructions. Co-precipitated proteins with biotin-labeled circRNA probes and control probes were further determined by Western blotting.

### Xenograft tumor model

Female BALB/c nude mice at 4–6 weeks old were obtained from Charles River Company (Beijing, China). One week prior to the experiment, ten nude mice were subcutaneously implanted with E2 pellets (0.72 mg/pellet; 60-day release). Then, the mice were randomly divided into two groups and subcutaneously injected with MCF7 cells stably overexpressing circRNA-SFMBT2 and control cells, respectively. The tumor size was measured every seven days with a vernier caliper starting when tumors became palpable, and the tumor volume was calculated as length × width^2^ × 0.5. Five weeks later, the mice were sacrificed, and then excised tumors were weighed (mg). Tumor tissues were processed for histological examination. All studies involving animals were performed in accordance with guidelines approved by the Animal Care and Use Committee of Shandong University.

### Immunohistochemistry (IHC)

Tissue samples were fixed in formalin, dehydrated through graded ethanols and xylenes, embedded in paraffin, and sectioned to 4 μm thickness. The sections were dewaxed by xylene and rehydrated with descending concentrations of ethanol. After antigen retrieval was performed with sodium citrate or EDTA solutions, sections were treated with 3% hydrogen peroxide and goat serum to block the endogenous peroxidase and nonspecific antigen binding sites, respectively. Followed by incubation with primary antibodies overnight at 4 °C, sections were washed with PBS, incubated with biotinylated secondary antibodies and streptavidin-conjugated horseradish peroxidase (HRP), stained using DAB chromogenic kit (ZSGB-BIO, Beijing, China), and counterstained using hematoxylin. After staining, sections were dehydrated with increasing concentrations of ethanol, cleared in xylene, and mounted with neutral gum before placing the coverslip. Lastly, photographs were captured using Leica light microscope.

### Statistical analysis

In this work, statistical analyses were performed using R project (Version 3.6.1) and GraphPad Prism 8. Statistical differences between two groups were determined by Student’s *t*-test, and between more than two groups by one-way ANOVA unless otherwise noted. Survival data was analyzed by Kaplan–Meier analysis. Statistical significance was set at a *p*-value < 0.05. Data were reported as the means ± SDs from three independent experiments.

## Supplementary information

Supplementary Materials

Reproducibility checklist

Western blot

## Data Availability

The datasets generated or analyzed during the present study are included in the article and its supplementary files.
