# Supplementary material for: circRNA-SFMBT2 orchestrates ERα activation to drive tamoxifen resistance in breast cancer cells
Source: Cell Death Dis. 2023 Jul 31;14(7):482. doi: 10.1038/s41419-023-06006-5 (PMC10390580; doi:10.1038/s41419-023-06006-5)
Supplement: Supplementary file 1 — Supplementary Materials [file 41419_2023_6006_MOESM1_ESM.docx]

**Supplementary Materials**

**Supplementary Tables**

**Table S1. Primer sets used for qPCR**

| **Primer set** | **Primers** | **Sequence(5’-3’)** |
| --- | --- | --- |
| circRNA-SFMBT2 | Forward | GCAGATTTGCGAAGCCATTTC |
|  | Reward | TCTGTGTACTTCTCTTTGATTGCT |
| SFMBT2 | Forward | TGGCAGCGACATCCTTAT |
|  | Reward | ATTACAACCTGGGCGACA |
| RNF181 | Forward | GACTGCCATTGAGATGCCTTGC |
|  | Reward | GCCTTATCTCGTCTGTGCTCCT |
| QKI | Forward | TCCGAGGCAAAGGCTCAATGAG |
|  | Reward | GCTCTGTTCTGAGCATCTTCCAC |
| QRE#1 | Forward | GGCAACTGAGTGAATGTT |
|  | Reward | TTCCAAATGTCCTCCTAC |
| QRE#2 | Forward | GACACTGCCACGTTATTC |
|  | Reward | CCAGACTCTTATCCCAAC |
| QRE#3 | Forward | TGGGATTATAGGCGTGAG |
|  | Reward | GCTACAAAGGGTAAGAAA |
| TFF1 | Forward | TTGTGGTTTTCCTGGTGTCA |
|  | Reward | TCGAAACAGCAGCCCTTATT |
| c-Myc | Forward | CCTGGTGCTCCATGAGGAGAC |
|  | Reward | CAGACTCTGACCTTTTGCCAGG |
| CyclinD1 | Forward | ATGGAACACCAGCTCCTGTG |
|  | Reward | ACCTCCAGCATCCAGGTGGC |
| CTSD | Forward | ATTCCCGAGGTGCTCAAGAA |
|  | Reward | TGCCAATCTCCCCGTAGTAC |
| β-actin | Forward | CATGTACGTTGCTATCCAG |
|  | Reward | CTCCTTAATGTCACGCACG |
|  |  |  |

**Table S2. Sequences for the nucleic acids used in this study**

| **Targets** | **Targeted Sequence (5’-3’)** |
| --- | --- |
| si1-circRNA-SFMBT2 | ACUAAGCAAUCAAAGAGAATT |
| si2-circRNA-SFMBT2 | GCGUCGGUGACUAAGCAAUTT |
| si-RNF181 | CAGCAACACCGACUGGAGATT |
| circRNA-SFMBT2 probe(FISH) | TGTACTTCTCTTTGATTGCTTAGTC |
| Sense probe(RNA pull-down) | TCGGTGACTAAGCAATCAAAGAG |
| Antisense probe(RNA pull-down) | CGCAGGAGAGATGTAAAAGGG |

**Table S3. Antibodies used in the study**

| **Antibody** | **Catalog Number** | **Company** | |  |
| --- | --- | --- | --- | --- |
| anti-ERα | ab16660 | Abcam |  |  |
| anti-RNF181 | 20408-1-AP | Proteintech group |  |  |
| anti-Flag | F2555 | Sigma-Aldrich |  |  |
| anti-HA | 51064-2-AP | Proteintech group |  |  |
| anti-Ubiquitin | sc-8017 | Santa Cruz Biotechnology |  |  |
| anti-Ubiquitin (K48) | ab140601 | Abcam |  |  |
| anti-Ubiquitin (K63) | ab179434 | Abcam |  |  |
| anti-Ki67 | 27309-1-AP | Proteintech group |  |  |
| Anti-rabbit IgG, HRP-linked Antibody | 7074 | Cell Signaling Technology |  |  |
| Anti-mouse IgG, HRP-linked Antibody | 7076 | Cell Signaling Technology |  |  |
| anti-β-actin | 3700 | Cell Signaling Technology |  |  |

**Supplementary Figures**

**Supplementary Figure 1**

**
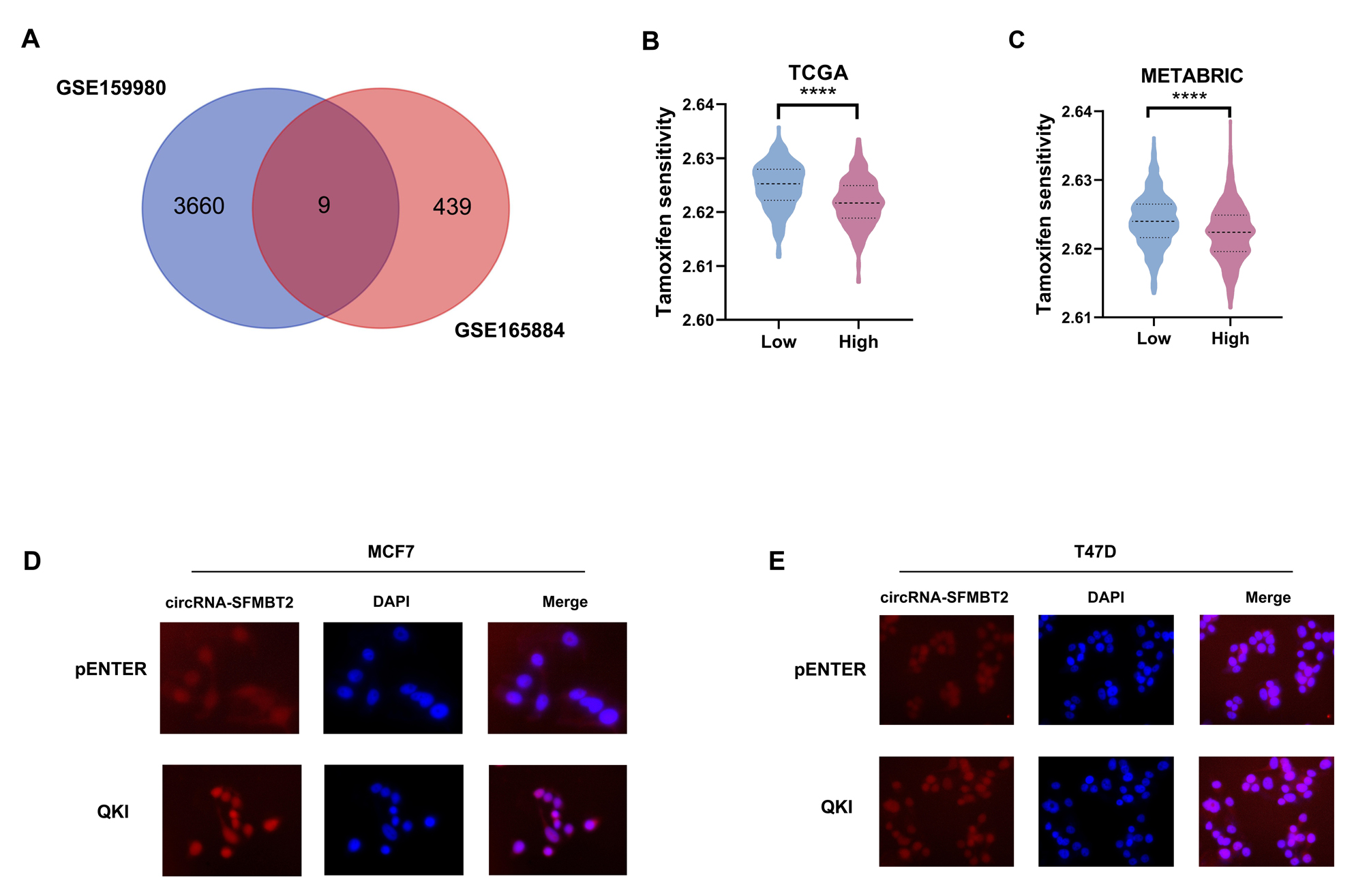
**

**Figure S1.** (A) Limma analysis was used to detect differentially expressed circRNAs in GSE159980 and GSE165884, of which 9 circRNAs were overlapped. (B, C) OncoPredict algorithm was applied to investigate the effect of circRNA-SFMBT2 on tamoxifen sensitivity in ER^+^ breast cancer patients from TCGA and METABRIC datasets. (D, E) RNA FISH was used to assess the effect of QKI overexpression on circRNA-SFMBT2 expression level in MCF7 and T47D cells.

**Supplementary Figure 2**

**
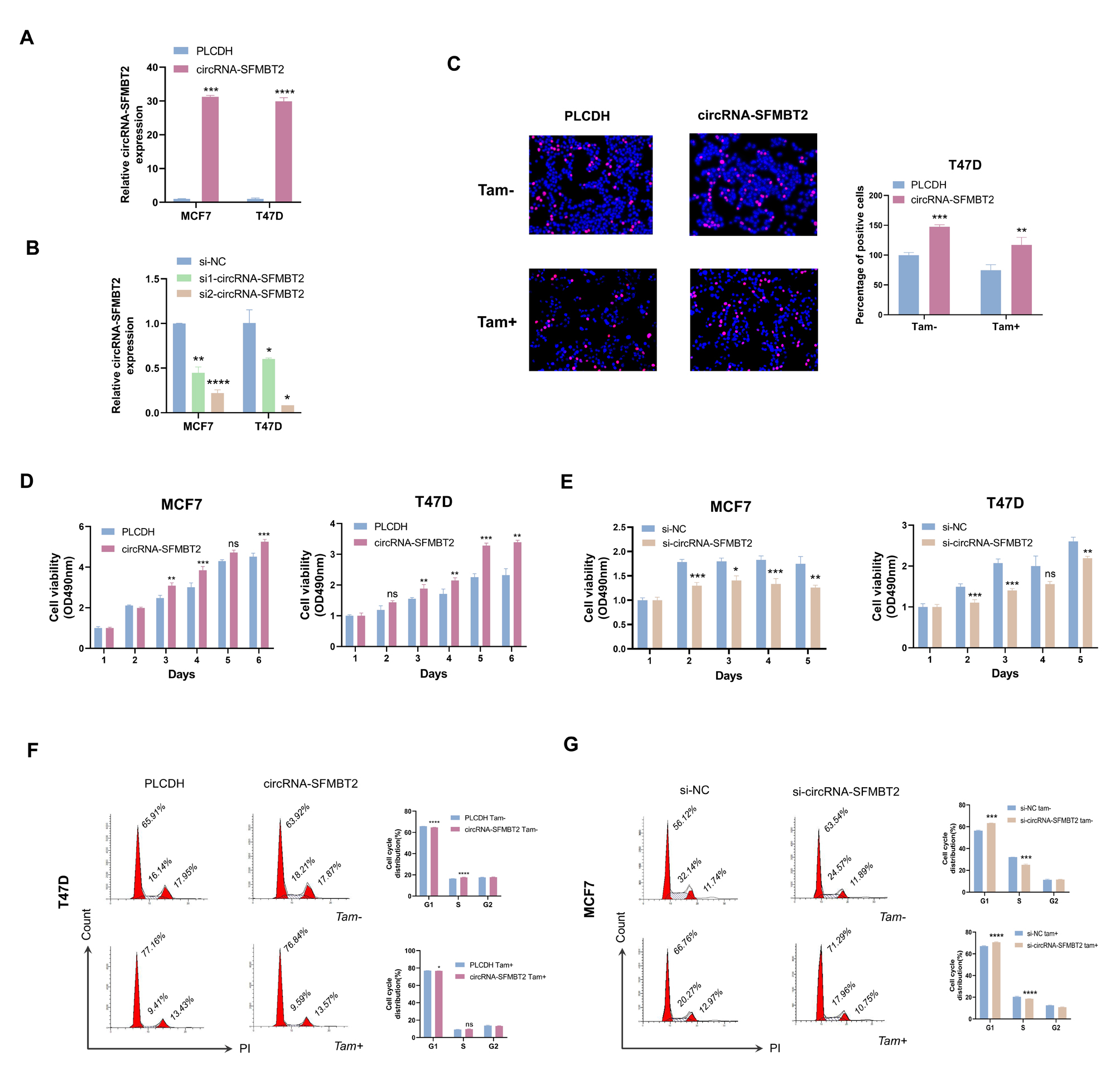
**

**Figure S2.** (A, B) qPCR assay was performed to evaluate the efficacy of transient transfection using circRNA-SFMBT2 overexpressing plasmids or siRNAs in both MCF7 and T47D cells. (C) EdU assay showing the effect of circRNA-SFMBT2 overexpression on the growth of T47D cells with or without the treatment of 5μM Tamoxifen (Tam). (D-G) Cells were transfected with circRNA-SFMBT2 siRNA or expression plasmid, and then treated with or without 5μM Tam. MTT assay showing relative cell growth at indicated times in the presence of 5μM Tam (D, E). Flow cytometry showing the cell cycle distribution (F, G).

**Supplementary Figure 3**

**
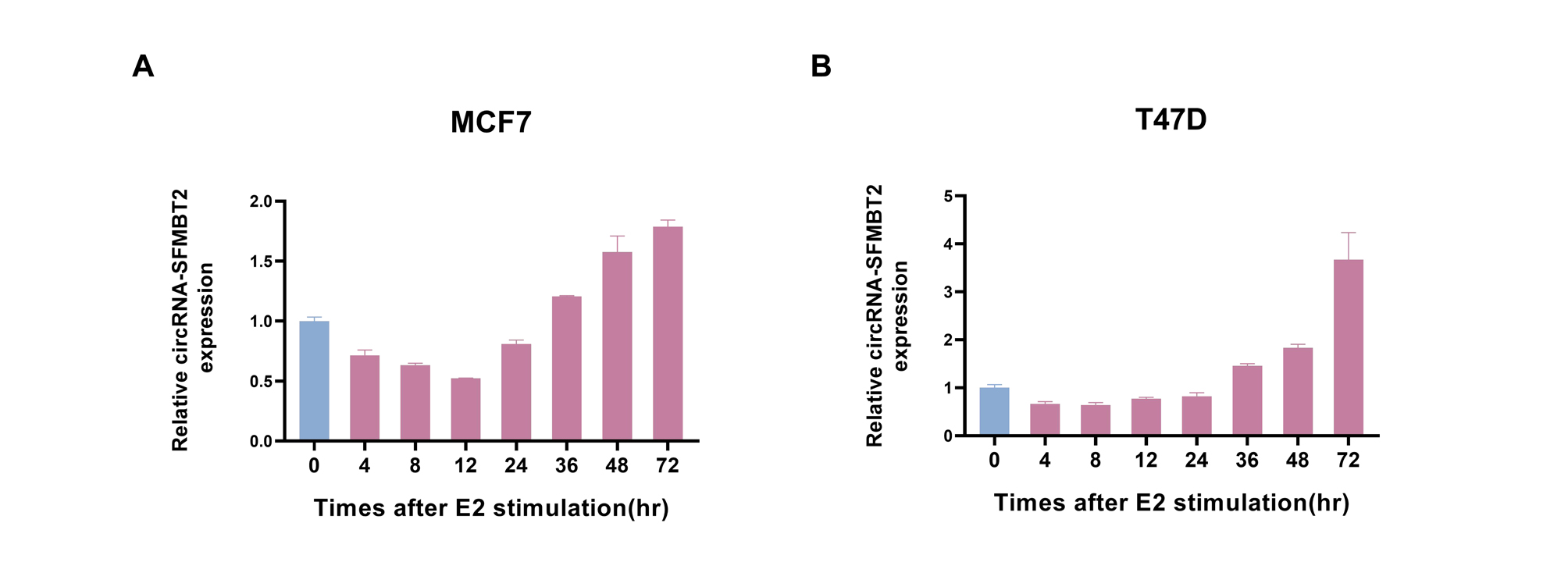
**

**Figure S3.** (A, B) MCF7 and T47D cells were hormone starved for 3 days and treated with 10nM E2 for 0, 4, 8, 12, 24, 48, and 72h. RNAs were collected and subjected to qPCR analysis for the detection of gene expression.

**Supplementary Figure 4**

**
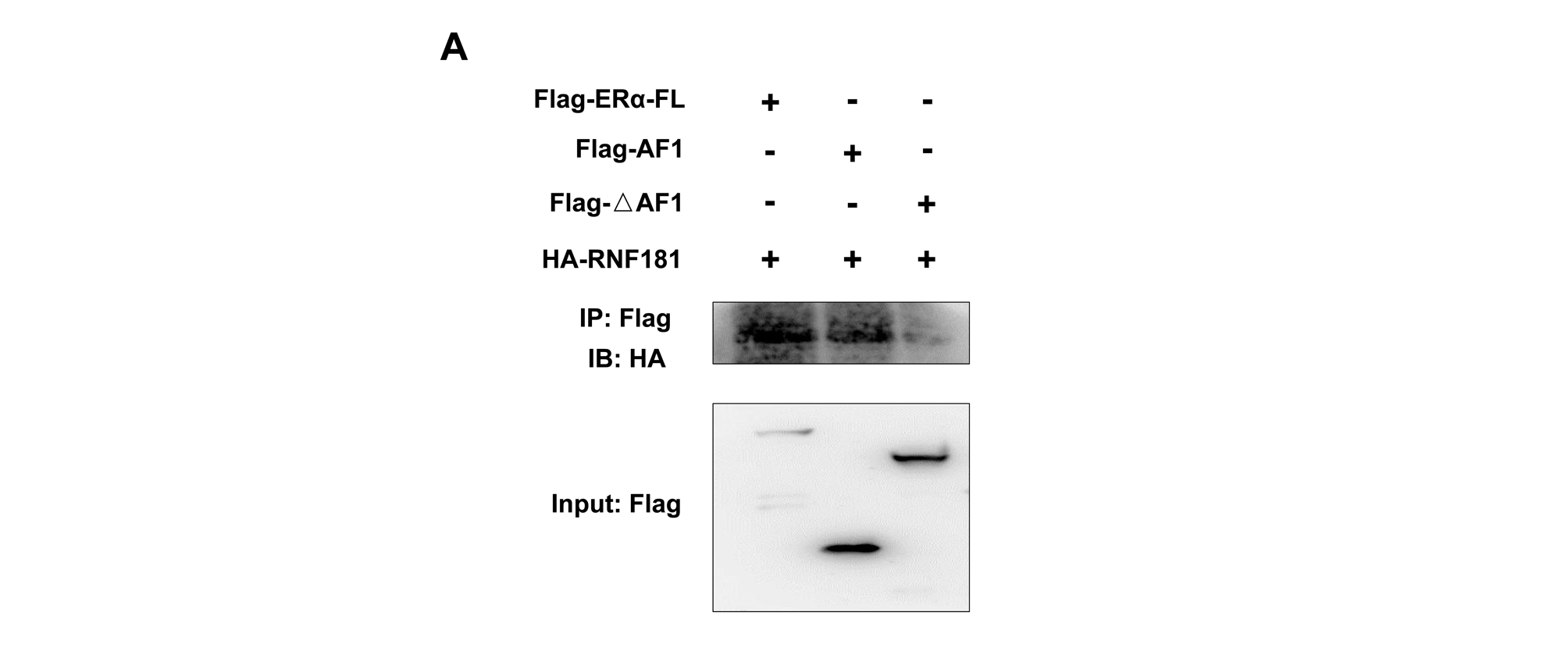
 Figure S4.** (A) Immunoprecipitation was used to determine the interaction of RNF181 with ERα.The results indicated that ERα can bind to RNF181 in the AF1-dependent manner.
