# Supplementary material for: circRNA-SFMBT2 orchestrates ERα activation to drive tamoxifen resistance in breast cancer cells
Source: Cell Death Dis. 2023 Jul 31;14(7):482. doi: 10.1038/s41419-023-06006-5 (PMC10390580; doi:10.1038/s41419-023-06006-5)

Unedited blots

Fig.4G

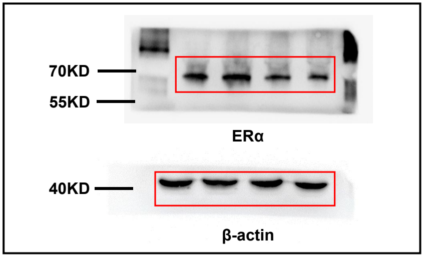

Fig.4H

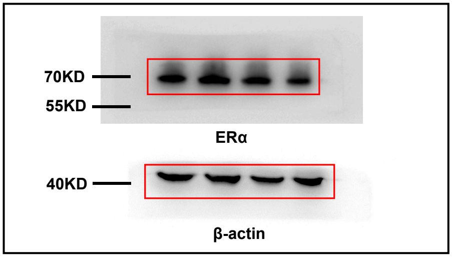

Fig.5D

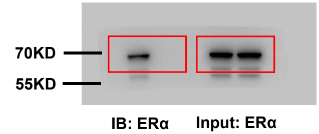

Fig.5F

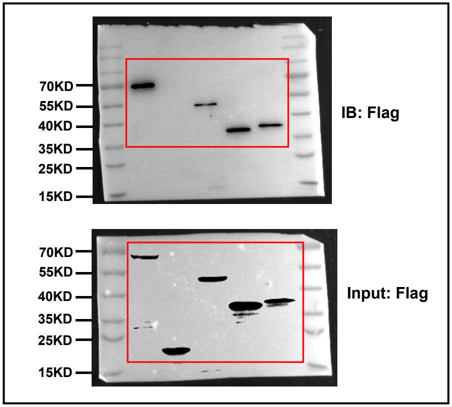

Fig.5H

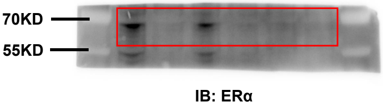

Fig.6B

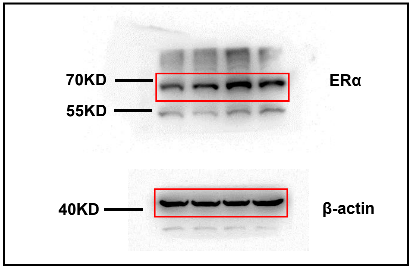

Fig.6C

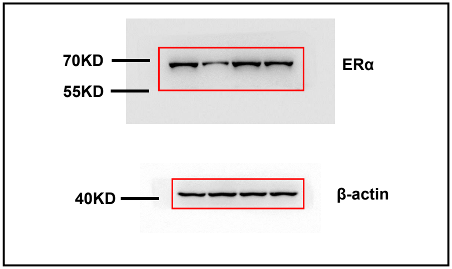

Fig.6D

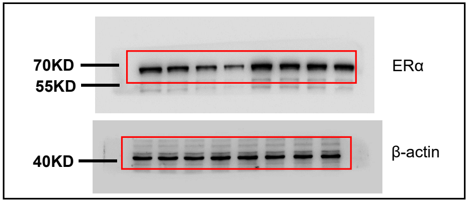

Fig.6E

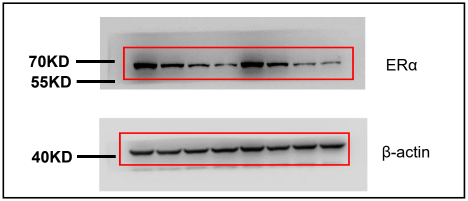

Fig.6F

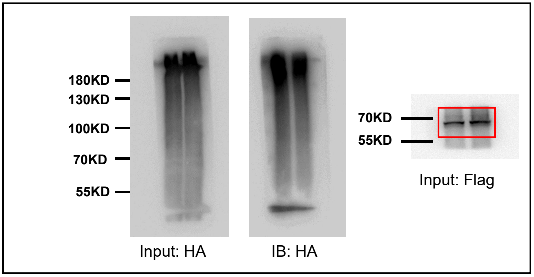

Fig.6G

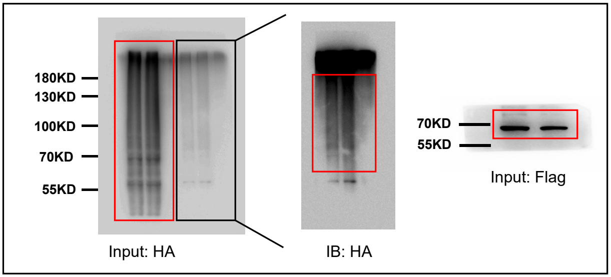

Fig.6H

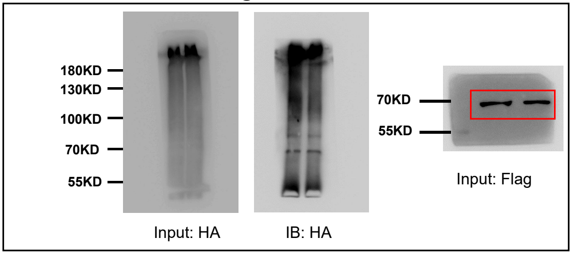

Fig.6I

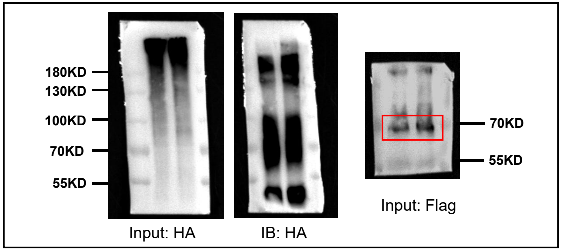

Fig.6J

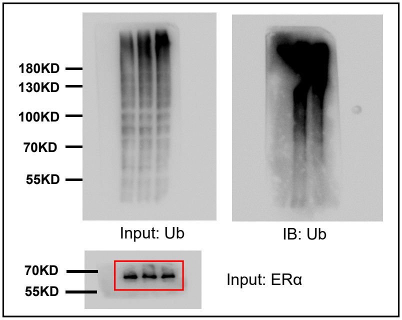

Fig.6K

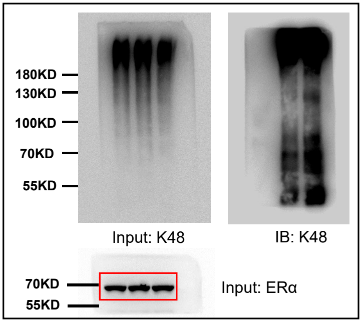

Fig.6L

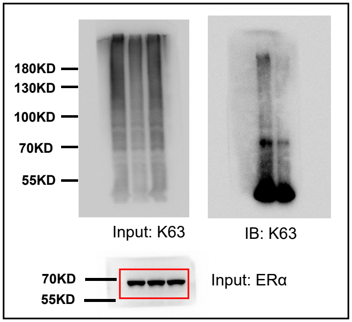

Fig.7H

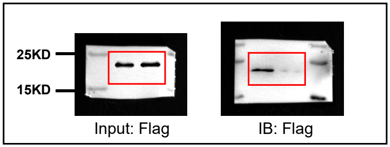

Fig.7K

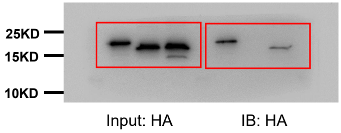

Fig.7L

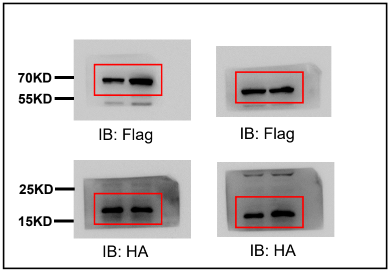

Fig.7M

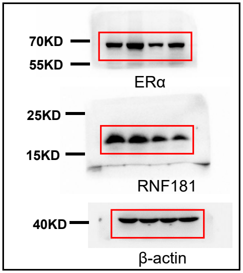

Fig.7N

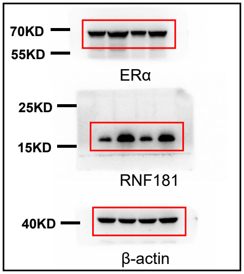

Fig.7O

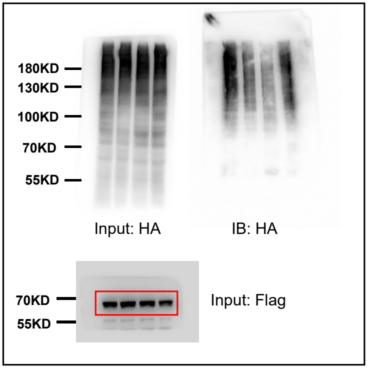

Fig.7P

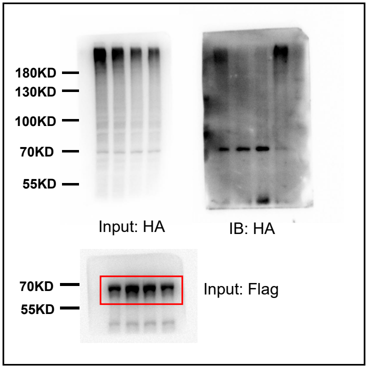

Fig.7Q

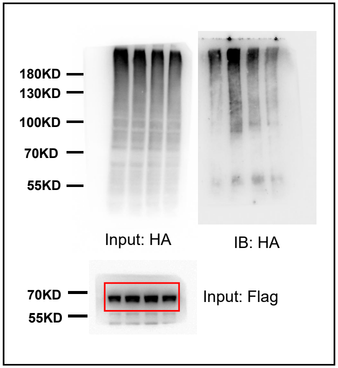

Supplement: Supplementary file 3 — Western blot [file 41419_2023_6006_MOESM3_ESM.pdf]
